# Integrating Disaster Response Tools for Clinical Leadership

**DOI:** 10.5811/westjem.35390

**Published:** 2024-11-21

**Authors:** Kenneth V. Iserson

**Affiliations:** The University of Arizona, Department of Emergency Medicine, Tucson, Arizona

## Abstract

**Background:**

Disastrous situations in the emergency department (ED) or community can overwhelm even the best-prepared teams due to their complexity and dynamic nature. In this paper we propose an integrated approach to disaster management, combining six theoretical and practical frameworks to enhance decision-making and operational effectiveness.

**Discussion:**

The approach begins with “sensemaking,” an instinctive process that helps leaders quickly gain situational awareness, a crucial foundation for the recognition-primed decision process (RPD). RPD enables swift, experience-based decisions without exhaustive analysis, aligning them with the appropriate domain in the Cynefin framework to guide subsequent interventions. In chaotic situations, rapid action is necessary, and the edge-of-chaos theory guides leaders to balance order and chaos for optimal adaptability. Complexity theory aids in managing the unpredictable elements of a crisis, highlighting the need for flexible responses. Finally, the Incident Command System ensures effective implementation by providing a standardized approach to command, control, and coordination. This cohesive strategy equips emergency physicians and incident commanders to manage both internal ED crises and broader community disasters effectively, with an emphasis on the importance of training in these frameworks to enhance the resilience of emergency medical services.

**Conclusion:**

This multifaceted approach should improve disaster management by better preparing responders for the unpredictable nature of emergencies, enabling effective evaluation and management of complex scenarios, and leading to a more rapid restoration of order.

## INTRODUCTION

Disasters, both natural and man-made, vary widely in scope and scale. They involve complex interactions between patients, the environment, and healthcare systems. Such incidents pose significant challenges to the entire emergency medical system (EMS) continuum, including emergency departments (ED), prehospital care systems, and disaster response teams.^1^ While emergency physicians (EP) are prepared to manage crises, their training may not extend to situations that lack available resources or that go beyond their knowledge or experiences.^2^

Disasters can be internal or external.^3^ Healthcare facilities may face internal disaster when treating patients with unusual or complex life threats, or when their normal functions are disrupted. Disruptions may result from the loss of essential resources such as water, power, computer systems, or staff; a sudden increase in patient numbers or severity; or imminent threats like fires, bomb threats, or active shooters. External disasters occur outside healthcare facilities and may be simple—typically short-lived and manageable using local resources or extended—causing widespread damage and injuries that necessitate external assistance to support the surviving local services, including healthcare facilities. Widespread disasters are long-term situations arising from pandemics, drought, famine, and war.^4^ Despite their differences, all disasters and disordered clinical situations share common elements that can inform effective management (Table 1). Managing a disordered situation as an EP or as a prehospital care or disaster team incident commander (IC) requires a comprehensive approach that addresses all these elements.

**Table 1.** Elements to address in all types of disasters.^5^ ^,^ ^6^

1. Strategic planning: Developing a comprehensive and flexible plan to address different types of disasters and disordered clinical situations.
2. Hazard assessment: Identifying potential hazards and assessing their potential impact to prioritize resources and develop appropriate responses.
3. Risk management: Identifying, assessing, and implementing measures to reduce risks.
4. Mitigation: Taking steps to reduce a disaster’s effect.
5. Preparedness: Ensuring that resources, systems and staff are ready to respond effectively when disasters occur.
6. Response: Taking measures to promptly evacuate people, provide medical care, and coordinate resources in a disaster situation.
7. Recovery: Making provisions to restore normalcy following a disaster.

## BACKGROUND

### Problem

This paper addresses the inadequacy of current disaster management approaches, which often rely on weak or inflexible evaluation and management tools. When used individually, these tools are often too rigid to adequately plan for, manage, and recuperate from the complex and unpredictable challenges posed by large-scale disasters. Most traditional disaster planning generates detailed plans that may fail when confronted by real-world scenarios. Since “no plan survives contact with the enemy,”^7^ disaster management fails when leaders rigidly follow a single method or plan that lacks the scope and flexibility offered by integrating multiple approaches.

Educational programs for disaster preparedness have been specifically designed for EPs, surgeons, intensivists, anesthesiologists, and similar medical specialties.^8^ These have included simulations, online games, case studies, and shared experiences with seasoned professionals. Yet it is unclear whether current systems to educate professionals about disaster management provide them with the adequate tools to deal with internal and external disasters.^9^ ^–^ ^11^ The integrated approach proposed in this paper equips EPs and ICs with comprehensive tools for managing both internal ED crises and wider community disasters. It has the flexibility to enhance decision-making in high-pressure situations and improve their coordination.

While already a small element of medical education, use of the methods that comprise the integrated approach should be expanded and integrated into residency and continuing medical education programs, as well as into training for potential ICs in EMS or fire services. Through experiential learning and scenario-based training, future leaders can develop the RPD skills necessary to navigate the complexities of disaster management, boosting their confidence and agility in emergencies.^12^

## OBJECTIVE

### Proposed Solution

In this paper we propose combining six disaster evaluation and management methods into a novel integrated approach that provides a structured methodology adaptable to changing circumstances. This fusion of multiple theoretical and practical frameworks provides flexibility and enhances emergency decision-making and operational effectiveness. The proposed framework includes, in order, the following:

1. Sensemaking
2. Recognition-primed decision-making (RPD)
3. Cynefin framework
4. Edge-of-chaos theory
5. Complexity theory
6. Incident Command System (ICS)

While several of the disaster assessment and management elements described in this paper are taught individually in disaster-oriented courses, others, such as the edge-of-chaos and complexity theories, have rarely been discussed in the healthcare literature.^13^ ^,^ ^14^

### Interconnections and Effectiveness

Each component in crisis management is interconnected, collectively contributing to the overall effectiveness of the process. The proposed sequence begins with sensemaking, which equips leaders with the situational awareness needed to quickly identify familiar patterns or cues. This awareness is crucial for RPD, a process that allows leaders to draw on past experiences to swiftly determine a course of action without exhaustively analyzing every possible option.

The decisions made through RPD are then aligned with the appropriate domain in the Cynefin framework, which helps determine the most effective approach to managing the crisis. If a situation falls into the chaotic domain, leaders must act rapidly to stabilize the environment. This is where leaders can use the edge-of-chaos theory to rapidly act at the brink of chaos, allowing for innovation and adaptability. Complexity theory then allows leaders to better understand and manage the unpredictable and interconnected elements of a crisis. Finally, the ICS ensures that these strategies are executed effectively by providing a standardized approach to command, control, and coordination. The ICS enables leaders to manage complex situations by organizing teams, delegating tasks, and ensuring clear communication.

Each component builds on the previous one, contributing to a cohesive and adaptive approach to managing critical, time-sensitive situations. The interplay between these theories and frameworks allows leaders to evaluate and manage complex scenarios effectively in crisis and disasters.

### Limitations of this Construct

The balance of this paper describes a logical progression of six assessment and managerial tools that EPs and ICs can use when dealing with ill-defined problems under high-stress conditions. Despite the presentation of these decision-making elements in sequential order, they are distinct components of a theoretical flowchart designed for disaster assessment and decision-making. This structured pattern will not always apply, since the real world is unpredictable and messy. Some steps may need to be used simultaneously; in other cases, previous steps will need to be revisited and adjusted as new information clarifies the situation.

## DISCUSSION

The following sections describe the six components of this proposed system in more detail, with information on how they build on each other to influence outcomes, whether they are currently taught, and best ways to introduce them into a curriculum.

### Component 1: Sensemaking in emergency medicine: initial assessment and situational awareness

In emergency medicine (EM) and disaster situations when information is often incomplete or rapidly evolving, the ability to quickly interpret confusing or unclear situations—known as sensemaking—is essential. Emergency physicians and ICs must swiftly collect information, identify patterns, formulate and then continually update plausible narratives based on direct observations and inputs from initial responders. Such input is essential for grasping the nature and scale of an event, since early misunderstandings can lead to ineffective or dangerous decisions.

Sensemaking is triggered when people encounter unexpected situations, those that contradict their expectations (eg, counterfactuals), or a significantly ambiguous event or issue.^15^ These situations may disrupt normal routines, leading individuals to question fundamental assumptions about how they should act. Emergency physicians typically engage in sensemaking when faced with diagnostic challenges where symptoms may not clearly indicate a specific disease, or in disaster management scenarios where information is inconsistent or incomplete. By asking appropriate questions (Table 2), they can construct a mental model from available data, begin to understand the unfolding situation, evaluate available resources, and specify immediate goals.^12^ ^,^ ^16^

**Table 2.** Sensemaking questions. Used to identify key stakeholders, evaluate available resources, and specify immediate goals in an unclear situation.

• Who is affected?
• What are the immediate threats to life and safety?
• What is the primary goal of the response effort (eg, rescue, evacuation, treatment)?
• What equipment, personnel, and supplies are on hand?
• What other resources are available?
• What more do we need to know to move forward?
After addressing these questions, the IC can then decide:
• What do I want to do?
• What do I have to do?
• What can I do?
• What am I trying to achieve?

*IC*, incident commander.

When a leader arrives at an emergency site, individuals such as nurses, housestaff, and first responders might already be working to stabilize the situation. These individuals often have a better understanding of the current circumstances than the newly arrived leader. It is important for leaders to acknowledge the value of the actions already in progress and to avoid interrupting the team’s momentum by stopping their activities for detailed briefings, if possible.^12^

Sensemaking emphasizes flexibility, allowing leaders to navigate the delicate balance between rigid adherence to protocols and the need for real-time adaptation. A key strategy within sensemaking is to initiate decision-making in disordered situations with targeted questions rather than immediately defaulting to established protocols like the ICS.^17^ This approach helps teams build a coherent narrative amid chaotic ambiguity (eg, multiple potential interpretations of a situation) and uncertainty (eg, a lack of interpretations that leaves individuals unsure of the next steps).

To address rapidly deteriorating situations, leaders must remain flexible and understand that traditional emergency response protocols, while valuable, may be difficult to adapt to the unpredictable nature of disasters. Continuing the sensemaking process enables leaders to shift their objectives as the situation develops (eg, from resuscitation to stabilization, rescue to recovery, search to evacuation). Sensemaking also aids in categorizing the situation using the Cynefin framework, a tool for managing complexity and uncertainty (Table 3).

**Table 3.** Progression from sensemaking to recognition-primed decision-making (RPD) and the Cynefin framework in disasters.

• Assess the scope and consequences of the disaster, including immediate life-threatening concerns and property damage.
• Identify critical tasks and determine their priority.
• Establish an understanding of the situation, focusing on plausibility rather than absolute precision.
• Recognize familiar elements within a complex situation to begin imposing order on chaos and guide the situation toward normalization.

### Component 2: Recognition-primed decision-making in emergency and disaster management

Leaders with experience in emergency and disaster management can use RPD to quickly assess complex time-sensitive situations. They leverage their store of experiences and tactics from previous incidents and training to select effective courses of action.^18^ ^,^ ^19^ Instinctively recognizing familiar elements, ie, sensemaking, they can use those insights to construct a reasonable understanding of the crisis.^19^

This pattern recognition enables them to make swift initial decisions to mitigate immediate threats. While not always fully nuanced, these decisions are crucial for stabilizing the situation and allowing time for more comprehensive data-gathering. The decision-making process involves asking critical questions to assess the disaster type, casualty numbers, environmental hazards, and available resources, while also clarifying the primary objectives of the response effort. Such clarity is essential for directing actions and resources toward meaningful outcomes.

### Component 3: Using the Cynefin framework in emergency and disaster management

The Cynefin framework, developed by Dave Snowden and Mary E. Boone, can enable EPs and ICs to quickly categorize and respond to situations based on their complexity and enhances their abilities to make swift, effective decisions under pressure.^20^ This model (Figure) helps differentiate among clear/obvious, complicated/difficult, complex, chaotic, and disarray/confusion/disordered/ uncertain domains, each of which necessitates a specific response strategy (Table 4) to restore order. As such, it provides an essential tool for managing the diverse and unpredictable nature of emergency scenarios. Leaders in a variety of healthcare fields have modified this model for various scenarios.^21^

**Figure.**
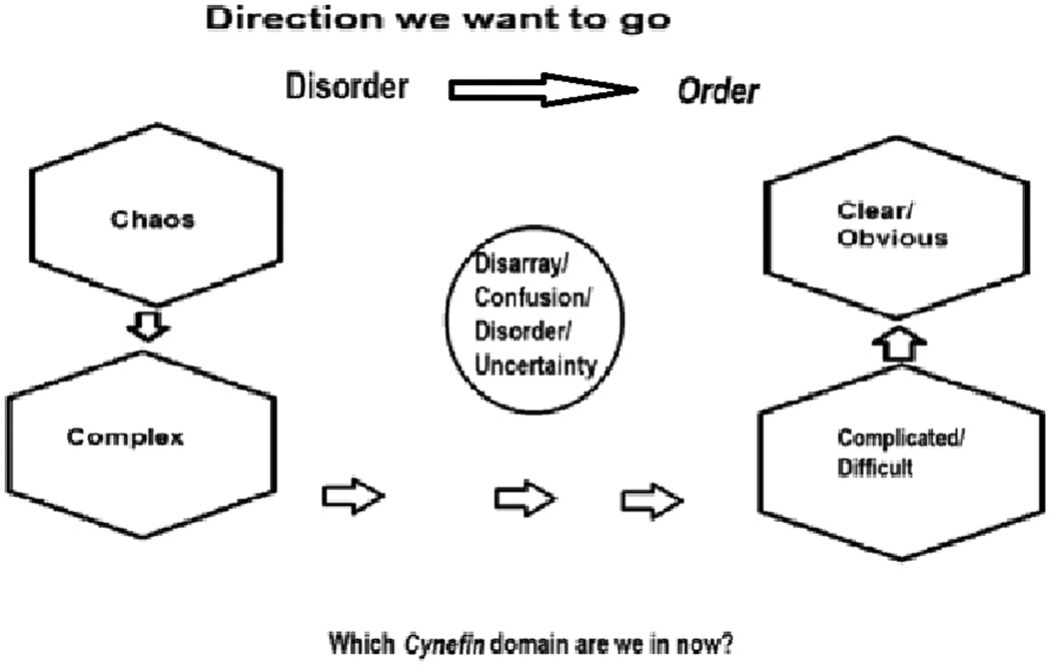
This Cynefin model illustrates the desired management flow from disorder to order. In disordered situations, the leader identifies the situation’s domain (chaotic, complex, complicated, or clear) and uses the correct response strategy (Table 4).

**Table 4.** Response strategies for five domains in the Cynefin framework.

• Simple/clear: Confirm rules or precedents, apply best practices.
• Complicated: Analyze the situation, apply expert knowledge.
• Complex: Experiment and adapt responses based on emergent patterns.
• Chaotic: Act immediately to stabilize, seek novel solutions.
• Disorder: Gather more information to categorize the situation correctly.

Originating in the business world, the Cynefin model has been adapted to provide clinicians with “a taxonomy for categorizing the various types of uncertainty, as well as a framework to apply when navigating uncertainty during clinical challenges. These tools can help students make sense of uncertainty and determine actions in a complex health system.”^22^ In situations categorized as simple or clear, where the relationship between cause and effect is straightforward, the strategy involves making sense of the situation by gathering data, confirming the problem has clear rules or precedents, and responding with established protocols such as the ICS. Such clear, predictable problems with well-understood solutions are amenable to best practices and standard operating procedures. For example, a patient with an isolated fractured humerus or minor injuries from a motor vehicle accident would fit into the “simple” domain, where established medical protocols are directly applicable.

More complex or chaotic scenarios demand innovative and adaptive strategies due to their unpredictability and the opaque relationship between cause and effect. For instance, a patient presenting with a rare combination of symptoms or a catastrophic event like a building collapse with multiple casualties would fall into these domains. Such situations require exploratory approaches that emphasize experimentation rather than linear solutions, which often prove insufficient (see Appendix).

Karl Weick, an organizational theorist, emphasizes the importance of categorizing unclear or novel situations within the Cynefin framework to clarify and address them effectively. This structured approach allows practitioners to identify the domain a situation belongs to—be it simple, complicated, complex, chaotic, or disorder—and tailor their decision-making and actions accordingly. In more complex or chaotic domains, where uncertainty prevails, further information gathering is necessary to determine an appropriate response strategy. Here, the strategy shifts toward maintaining a balance between stability—using proven methods—and flexibility—adapting to the unique challenges of the situation, as suggested by the edge-of-chaos paradigm.

### Component 4: Navigating the edge-of-chaos paradigm in disaster response

The edge of chaos is a concept derived from complexity theory, which describes the precarious state existing between order and complete randomness.^23^ ^,^ ^24^ Within the context of the Cynefin framework, this concept represents an initial state of disorder, confusion, and uncertainty, where the application of sensemaking and RPD is crucial. This concept can be visualized as an infinitesimally thin membrane marking the transition from chaotic disorder to an ordered, manageable scenario. In such conditions, EPs and ICs must navigate a delicate balance between maintaining stability (order) and adapting to new information (flexibility). The EPs and ICs need to identify when an incident is at or nearing the edge of chaos to foster effective, adaptive responses to rapidly evolving conditions. They should be able to recognize the situational characteristics that indicate that they are approaching the edge of chaos. These include the following:

#### Increasing information complexity

This may involve contradictory reports, escalating numbers of casualties, or conflicting operational directives, all of which complicate the response effort.

#### Decision-making difficulties

As uncertainties and complexities multiply, the decision-making process becomes more challenging. The decline in effectiveness of standard protocols may indicate the need for adaptive, creative solutions.

#### Increased communication breakdowns

Effective communication is essential for maintaining order. Failures in technology or misunderstandings among team members that cause communication breakdowns may necessitate a shift in management tactics.

#### Resource limitations

A decrease in available resources, whether personnel, equipment, or information, can signal movement toward chaotic conditions. Monitoring resource usage and forecasting future needs are critical for recognizing and responding to this shift.

The edge-of-chaos paradigm underscores the complexity of decision-making under conditions characterized by time pressure, dynamic changes, and challenging logistics. It serves as a bridge across various disaster leadership methodologies, emphasizing the need to maintain a balance to prevent the situation from tipping into chaos. This approach requires EPs and ICs to be exceptionally agile, using a blend of established protocols and innovative solutions to manage disasters effectively. By recognizing and responding to the signs of the edge of chaos, leaders can prevent further deterioration of the situation and guide their teams toward restoring order and stability.

### Component 5: Complexity theory and its importance in emergency response

The EP often operates in conditions marked by high uncertainty and unpredictability, conditions that point to the need for an understanding of complexity theory. This theory, sometimes referred to as “high-dimensional chaos,”^25^ is rooted in mathematics, physics, and cellular biology, and can help EPs^26^ navigate the nature of emergencies, facilitating better long-term planning and resource allocation.^25^ It is a vital framework for enhancing decision-making, efficiency, and adaptability in the dynamic, unpredictable environments with multiple interconnected components typical of emergency and disaster responses.^27^ ^,^ ^28^

Although defining complexity theory has proven elusive and has rarely been applied to healthcare,^29^ ^,^ ^30^ it can help EPs discern whether a situation is complicated, with clear cause-and-effect relationships; complex, characterized by emergent behaviors; or chaotic, with unpredictable elements.

The core features of complexity theory in emergency response include unpredictability, self-organization, emergent behaviors, and feedback loops.

#### Unpredictability (nonlinearity)

In complex systems, the relationship between cause and effect is not linear, making outcomes hard to predict. Small changes can trigger disproportionately large effects, reflecting the system’s sensitivity to initial conditions (the “butterfly effect”).^27^ ^,^ ^28^ ^,^ ^31^ This aspect is crucial in EM, where a small alteration in team dynamics or decision points can significantly shift the outcome.

#### Self-organization

Complex systems exhibit a capacity to organize spontaneously without external control.^32^ In EM, this is observed when multidisciplinary teams (eg, a “flash mob”) rapidly assemble in response to a trauma call. Although their communications, equipment, command structure, and goals may not initially gel, they quickly align to function as a single organic team. Some volunteer wilderness rescue groups also work like this; knowing each other’s capabilities, they automatically place the most expert person at each stage in the lead role (eg, organizing the team, setting up technical gear, medical assessment/treatment, and evacuation) in what they term a “flowing command system.” [Personal communication, from the Southern Arizona Rescue Association, Pima County, AZ; February 1, 2024.]

#### Emergent behaviors

These behaviors, which are not predictable from the system’s initial conditions, often arise in complex situations. They may manifest as new patterns of team interaction or the use of innovative problem-solving approaches when standard procedures prove insufficient.^33^ As complex systems self-organize, they become more than the sum of their parts.^28^ This makes it impossible to precisely determine outcomes from interventions (nonlinearity).^34^

#### Feedback loops

Feedback is essential for adapting to changing conditions within emergency responses. These self-regulating systems can amplify or dampen the effects of the system’s behavior, enhancing adaptability and resilience.^35^ ^,^ ^36^ Managing complex situations requires leaders to know which interventions have succeeded. This allows teams to rapidly implement alternative actions when necessary. Complexity theory not only provides a theoretical framework but also sets the stage for practical decision-making in emergency situations. It offers insights into the nature of disorder and reminds leaders that unexpected behaviors and outcomes are typical under complex conditions. Furthermore, the theory underscores the potential for order to emerge from chaos through self-organization and resilience.

Understanding the interactions among system components—individuals, teams, resources, and environmental factors—is key to managing overall system behavior. These interactions often give rise to new characteristics (ie, emergent properties) that cannot be predicted from the system’s individual components alone. In essence, the sum of the parts is more than the whole. This requires managers to be both flexible and innovative.

Including complexity theory in emergency response training and operations enhances the ability of EPs and ICs to better manage disasters by preparing EPs to anticipate and respond to unexpected situations. Moreover, complexity theory supports the creation of networks that foster collaboration across various agencies and disciplines, optimizing the collective response effort. By embracing a flexible, adaptive mindset and implementing continuous learning and improvement, emergency professionals can navigate the evolving dynamics of emergencies more effectively (Table 5).

**Table 5.** How complexity theory has been used in mass disasters.

Incident	Complexity theory principle applied	Outcome
2017 Las Vegas shooting^37^	Feedback loops and interconnectivity	Rapid setup of coordinated triage areas led to efficient patient management despite overwhelming numbers.
Hurricane Katrina^38^	Emergence and adaptability	Ad-hoc medical hubs and trained leaders improved survival rates amidst infrastructure collapse and multiple leadership failures at all levels.
COVID-19 pandemic^39^	All principles of complexity theory	Dynamic response strategies like adaptable hospital zones and real-time data tracking helped manage patient loads effectively.

### Component 6: Integration of the Incident Command System in EM and disaster response

After devasting wildfires in the 1970s, Southern California firefighters developed the ICS to respond to disasters in a coordinated manner and to request regional resources as the situation expanded. When the September 11 attacks showed that coordinating federal and state with local resources was vital, this was incorporated into the National Incident Management System.^40^ The ICS is an essential structured approach that integrates sensemaking and the Cynefin framework to manage resources effectively across various emergency scenarios. This system provides EPs and ICs with a standardized method for incident management that remains consistent regardless of the complexity of the situation—be it simple, complicated, complex, or chaotic. It ensures clear lines of authority, effective communication, and coordinated resource allocation, making it indispensable for managing both resources and personnel.

The ICS promotes effective coordination and communication among all responders, medical personnel, and rescue teams through its unified terminology, modular organization, and clarity in communication channels. These features are crucial, especially in complex situations involving multiple agencies, when it is necessary to have all parties aligned and informed. Despite its strengths, the ICS can be restrictive during the initial phase of disasters. The system’s reliance on checklists and tasks may lead first responders to prioritize procedural adherence over gaining a deeper understanding of the broader crisis. This adherence to protocol can limit the ability to address chaotic situations where no straightforward guide exists, as noted in the critique that “While everyone can be … trained to follow a checklist during later stages of an event, there is no exact guide for how to work through chaos.”^12^ ^,^ ^17^

As scenarios transition from chaotic and disordered states to more complex or complicated domains, the advantages of the ICS become more evident. In these stages, the structured nature of the ICS supports methodical decision-making, particularly in scenarios familiar to responders. It allows for deliberate actions based on thorough situational awareness and a factual understanding of the incident. Described as a military-type system emphasizing command, control, and coordination, the ICS is particularly suited for large-scale operations. Its structured approach is critical in guiding efforts in EM and disaster response, especially when combined with the principles of complexity theory. This integration acknowledges the dynamic and multifaceted nature of such environments, enhancing the system’s effectiveness.

By recognizing the strengths and limitations of the ICS within the framework of complexity theory, emergency management can adapt the system to better meet the demands of diverse emergency situations. This adaptation allows for a balance between the need for structured responses in familiar scenarios and the flexibility required in unpredictable or novel emergencies.

### Integrating educational methods for advanced decision-making frameworks into EM

Training EPs in advanced decision-making frameworks involves a structured approach to understanding and applying the various theories and systems crucial for effective emergency response. For the RPD model, scenario-based training is instrumental, allowing physicians to rapidly assimilate past experiences with current situations to make effective decisions under pressure. Integrating sensemaking into training involves scenario-based learning where physicians interpret complex or ambiguous information to construct a coherent understanding of evolving situations (Table 6).^41^ ^–^ ^43^

**Table 6.** Key outcomes of disaster management training for emergency physicians and incident commanders.

Outcome	Description	Impact on emergency care
Enhanced decision-making^44^	Ability to make informed decisions amidst chaos.	Leads to better patient outcomes and less confusion during crises.
Improved crisis management^45^	Effective management of sudden, unpredictable events.	Reduces errors and improves overall emergency response effectiveness.
Increased system resilience^46^	Strengthening of the emergency care system to withstand pressures.	Ensures sustainability and reliability of emergency medical services.
Proactive problem solving^47^	Anticipating and addressing complications before they escalate.	Decreases incident severity and improves patient recovery rates.
Team cohesion and dynamics^48^	Improved teamwork under stress through understanding of roles and dependencies.	Enhances operational efficiency and staff morale during emergencies.

Rusnack et al described a workshop to prepare clinicians to use sensemaking and the Cynefin framework to work through complex clinical challenges. Participants learned to categorize problems into simple, complicated, complex, or chaotic domains, and to apply appropriate decision-making strategies based on the situation’s nature.^39^ Complexity theory and the edge-of-chaos theory should be integrated into the curriculum through interactive lectures and group discussions that explore the dynamics of complex systems and their implications on emergency management.

The ICS, essential for managing emergency responses, requires both knowledge-based learning and practical exercises. Training should include detailed modules on ICS protocols and roles, supplemented by drills that simulate multi-agency response scenarios to foster coordination and communication. Training that blends theoretical instruction with practical exercises provides EPs with the skills needed to handle a wide range of challenges in emergency situations. This training leverages a deep understanding of different decision-making frameworks to improve their effectiveness and adaptability during real-world crises.

## CONCLUSION

This proposal to reformulate and integrate disaster evaluation methods will, hopefully, be a stimulus to emergency and disaster medicine to reconsider their approaches to crises because it does the following:

• Presents an innovative and integrated approach that addresses significant gaps in disaster management.
• Enhances decision-making, promotes comprehensive training, and improves emergency response effectiveness and outcomes.
• Significantly improves outcomes by preparing responders for the unpredictable nature of emergencies. By enhancing coordination, decision-making, and training, it builds more resilient EMS and optimizes resource allocation during crises.
• Lays the groundwork for future research to evaluate the combined framework’s effectiveness in real-world disaster scenarios. By presenting this concept, we can stimulate discussion and collaboration among emergency clinicians, researchers, and policymakers to advance the field of disaster management.

This exploration of disaster-management tools demonstrates that integrating multiple theoretical frameworks and methodologies, such as sensemaking, the recognition-primed decision process, the Cynefin framework, the edge-of-chaos theory, complexity theory, and the Incident Command System, can significantly enhance the decision-making process and operational efficiency in emergency and disaster management. This synthesis offers emergency physicians and incident commanders a robust toolset with which to navigate the multifaceted nature of disaster situations—whether these occur within healthcare facilities or in external environments.

By adopting this integrated approach, emergency management personnel can better address the inherent complexities of disaster scenarios, ensuring more rapid stabilization of chaotic situations and a more organized transition to recovery phases. The emphasis on dynamic and adaptable response strategies aids in managing emergencies where traditional, rigid command structures may fall short.

Furthermore, the paper highlights the critical role of continuous training and education in these integrated frameworks. It argues that well-prepared emergency teams, versed in these frameworks, are better equipped to manage the unforeseen and rapidly evolving challenges of disaster scenarios. The application of these comprehensive strategies not only enhances the capability of emergency personnel to perform under pressure but also improves overall patient outcomes and system resilience.

As disasters continue to present unique and complex challenges, ongoing research and adaptation of these frameworks will be essential. The evolving nature of global threats—ranging from natural disasters to technological and biological hazards—demands a progressive approach to emergency management education and practice. Integrating these diverse frameworks into a unified operational strategy ensures a well-rounded response capability, fostering a more resilient and effective EMS. This paper serves as a call to action for emergency response organizations and training institutions to embrace this multifaceted approach. It is vital for enhancing the preparedness and response effectiveness in the face of disasters.

## Supplementary Information




