# Supplementary material for: Integrating Disaster Response Tools for Clinical Leadership
Source: West J Emerg Med. 2024 Nov 21;26(1):30–9. doi: 10.5811/westjem.35390 (PMC11908526; doi:10.5811/westjem.35390)
Supplement: Supplementary file 1 [file wjem-26-30-s001.docx]

**APPENDIX: Case example: managing a crisis—from disorder to simple**

The following vignette illustrates how leadership in an academic ED applied several decision-making theories and frameworks to navigate through a critical incident involving mass exposure to a nerve agent. The incident demonstrates the sequential use of key tools: sensemaking and RPD, the Cynefin Framework, complexity theory, edge-of-chaos theory, and the ICS. Note: This scenario occurred before cellphones were commonly used.

Disorder: Sensemaking and Recognition-Based Decision Making

Upon receiving a distorted radio transmission about a middle school incident involving the nerve agent malathion, the ED was thrust into disorder. With limited information, the staff was uncertain and confused. The attending EP initiated RPD by quickly calling in an experienced EP to assist, leveraging his expertise to gain clarity and start the sensemaking process. This step was crucial in identifying the nature of the crisis and beginning to form a strategic response.

Chaos: Application of the Cynefin Framework

As the experienced EP arrived, the situation was chaotic with no clear information on the number of casualties or their conditions. Utilizing the Cynefin Framework, the EP classified the situation as ‘chaotic’ where rapid, decisive action was needed. He directly communicated with the 911 dispatch center and the nearby ED to gather critical information; this move had never been attempted before but proved essential in transitioning from chaos to a more structured approach.

Complex: Leveraging Complexity Theory

With new information, the complexity of the situation became apparent—approximately 200 children were exposed to the nerve agent, but none were critically affected. Using principles of complexity theory, the EP managed the numerous interconnected elements of the crisis. He orchestrated a large-scale response, including clearing the ED for incoming patients, setting up decontamination stations, and coordinating with various hospital departments. This approach emphasized understanding the interdependencies within the hospital and the broader emergency response system.

Complicated: Navigating the Edge of Chaos

As preparations unfolded, the scenario transitioned to a complicated domain where the relationships between actions and outcomes were not straightforward but could be deduced through analysis and expertise. The EP utilized the edge-of-chaos theory to maintain a balance between rigid control and adaptive flexibility. This was demonstrated by setting up specialized decontamination zones and organizing resources in a way that could be quickly adjusted as the situation developed.

Simple: Implementation of the ICS

Finally, as the buses with the affected children arrived, the situation moved into the ‘simple’ domain of the Cynefin Framework. The established ICS, which was integrated into the ED’s management protocols, facilitated a structured and efficient triage process. Clear protocols were followed as the EP reassured and directed the children through an improvised decontamination process and reunited them with their parents in an area staffed with chaplains, pediatricians and other support personnel. This step used established best practices in a stable environment where the inputs and expected outputs were well understood.

Case Resolution

The effective management of this emergency was a result of the strategic application of various decision-making tools and theories in a sequential manner. Each phase of the response was characterized by specific challenges that required different approaches, from gathering information and rapid decision making to complex coordination and executing well-established procedures. This case study exemplifies how EDs can enhance their response capabilities by integrating diverse theoretical frameworks into their operational protocols, ultimately improving outcomes in dynamic and complex emergency scenarios.
